# How do Japanese medical students use social media? A large multicenter survey of platform preferences and career decision-making

**DOI:** 10.1007/s00595-026-03328-7

**Published:** 2026-06-04

**Authors:** Takehito Yamamoto, Koya Hida, Shin Ishihara, Soichi Murakami, Yusuke Yamane, Tatsuya Kinjo, Misako Kinoshita, Kenta Horimukai, Atsushi Otsuka, Hideki Takami, Kazutaka Obama

**Affiliations:** 1 Department of Surgery, Graduate School of Medicine, Kyoto University, 54 Shogoin- Kawara-cho, Sakyo-ku, Kyoto, 606-8507 Japan; 2 Department of Gastroenterological Surgery, Fujita Health University, Toyoake, Japan; 3 Department of Gastroenterological Surgery II, Graduate School of Medicine, Hokkaido University, Sapporo, Japan; 4 Department of Surgical Oncology, Graduate School of Biomedical Sciences, Nagasaki University, Nagasaki, Japan; 5 Department of Digestive and General Surgery, Faculty of Medicine, University of the Ryukyus, Okinawa, Japan; 6 Department of Pediatrics, Jikei University Katsushika Medical Center, Katsushika, Japan; 7 Department of Dermatology, Kindai University Hospital, Osaka, Japan; 8 Department of Surgery, Graduate School of Medicine, Nagoya University Hospital, Nagoya, Japan

**Keywords:** Social media, Medical students, X, Twitter, Instagram, YouTube, Facebook, TikTok

## Abstract

**Purpose:**

Social media has emerged as an important tool for disseminating information to medical students; however, their platform preferences and usage purposes remain unclear.

**Methods:**

We conducted a multicenter web-based survey of medical students from eight Japanese medical schools in 2025, assessing their overall social media use, platform preferences by purpose, daily usage time, and the perceived usefulness of social media for career decision-making.

**Results:**

A total of 1,515 students participated in this study. YouTube (71.6%), Instagram (65.1%), and X (43.7%) were frequently used platforms, whereas Facebook (1.9%) was rarely used. Platform use differed by academic year: YouTube use was significantly higher among early-year students, whereas X use was significantly more common among advanced-year students (*P* = 0.043 and *P* < 0.001, respectively). Overall, 763 respondents (50.4%) perceived social media as useful for career decision-making, with X being used most frequently for this purpose, followed by YouTube and Instagram; this perception was significantly more common among the early-year students (*P* = 0.005).

**Conclusions:**

These findings provide valuable insights for educators and professional organizations seeking to engage medical students through social media. Understanding these trends is especially important as Japan faces a shortage of surgeons.

.

## Introduction

Medical students use different methods to obtain information. Traditional methods such as announcements sent through mailing lists are less likely to reach students, and posting information only on the websites of academic societies or universities is often ineffective, as unlike senior physicians, many students do not visit these sites regularly. In this context, social media has emerged as an important alternative tool for disseminating information to medical students [1].

The declining number of surgeons has become a serious issue in Japan. A recent nationwide online survey of 1,335 hospitals by the Japan Surgical Society revealed that 54.9% of teaching hospitals reported a shortage of surgeons, and only 22.2% considered the number of surgeons to be satisfactory [2]. To address this challenge, academic societies and medical schools have introduced various programs to encourage medical students to consider surgical careers. In this context, social media has become a critical promotional tool for medical students.

The Japan Surgical Society launched its official Facebook and X (formerly Twitter) accounts in February, 2024, and the Japanese Society of Gastroenterological Surgery launched its official X account in March, 2024. In addition, more than 30 surgical departments at medical schools throughout the country use Instagram to post information related to medical education and scientific research. However, it remains unclear which social media platforms medical students use and for what purposes.

Most previous reports investigating social media use for education and recruitment have been limited to evidence from the US [3–12]. There seems to be a substantial discrepancy between countries, and little knowledge is available about Japanese medical students. Learning about the current trends in social media use is important not only for educators in Japan but also for those in other countries who need to disseminate information to Japanese students. Thus, we conducted the present study to investigate the social media use of medical students in eight Japanese universities via a web-based questionnaire survey.

## Methods

### Study design and participants

This was a multicenter, cross-sectional survey conducted in 2025 among first- to sixth-year undergraduate medical students at five public and three private Japanese universities: Fujita Health University, Hokkaido University, Kyoto University, Nagasaki University, University of the Ryukyus, Jikei University School of Medicine, Kindai University, and Nagoya University. According to the study objective, an online questionnaire was developed using Google Forms.

## Survey content

The questionnaire collected information on patterns of social media use. Participants were asked to identify the social media platform they used most frequently overall, as well as the platforms used primarily for specific purposes, including medical or academic learning, entertainment, and social interaction. For overall social media use, respondents could select multiple platforms, whereas for each specific purpose, they were asked to select a single platform from a predefined list or indicate that they did not use any social media platforms. This list included the following social media platforms: X, Facebook, Instagram, TikTok, YouTube, and LinkedIn. Participants were also asked to report their primary purpose for using social media by choosing from categories such as learning, news consumption, entertainment, social interaction, or other purposes.

To assess differences in social media use by year of study, comparisons of each platform use between early-year (first- to third-year) and advanced-year (fourth- to sixth-year) medical students were conducted in addition to the proportion of students by individual years of study. To assess the role of social media in career development, respondents were asked whether they considered social media useful for their career choice. Those who answered “Yes” were then asked to identify the platform they found most useful for this purpose. Daily time spent on social media was assessed using predefined categorical ranges (< 1 h, 1–3 h, 3–5 h, and > 5 h).

## Ethical considerations

Responses were collected anonymously and no personally identifiable information was obtained. At the beginning of the survey, participants were informed that their responses would be anonymized and used solely for research purposes. Students who declined to participate were excluded.

This study was approved by the Kyoto University Graduate School and Faculty of Medicine Ethics Committee (reference no. R4577). The study design and manuscript preparation conformed to the Strengthening the Reporting of Observational Studies in Epidemiology (STROBE) Reporting Guidelines.

## Statistical analyses

Categorical variables are presented as absolute numbers and percentages. The chi-square test was used to compare categorical variables. Statistical analyses were performed using JMP Pro, Version 17 (SAS Institute Inc., Cary, NC, USA) and GraphPad Prism ver. 10 (GraphPad Software, Inc., San Diego, CA, USA).

## Results

### Participant characteristics and overall social media use

**Fig. 1:**
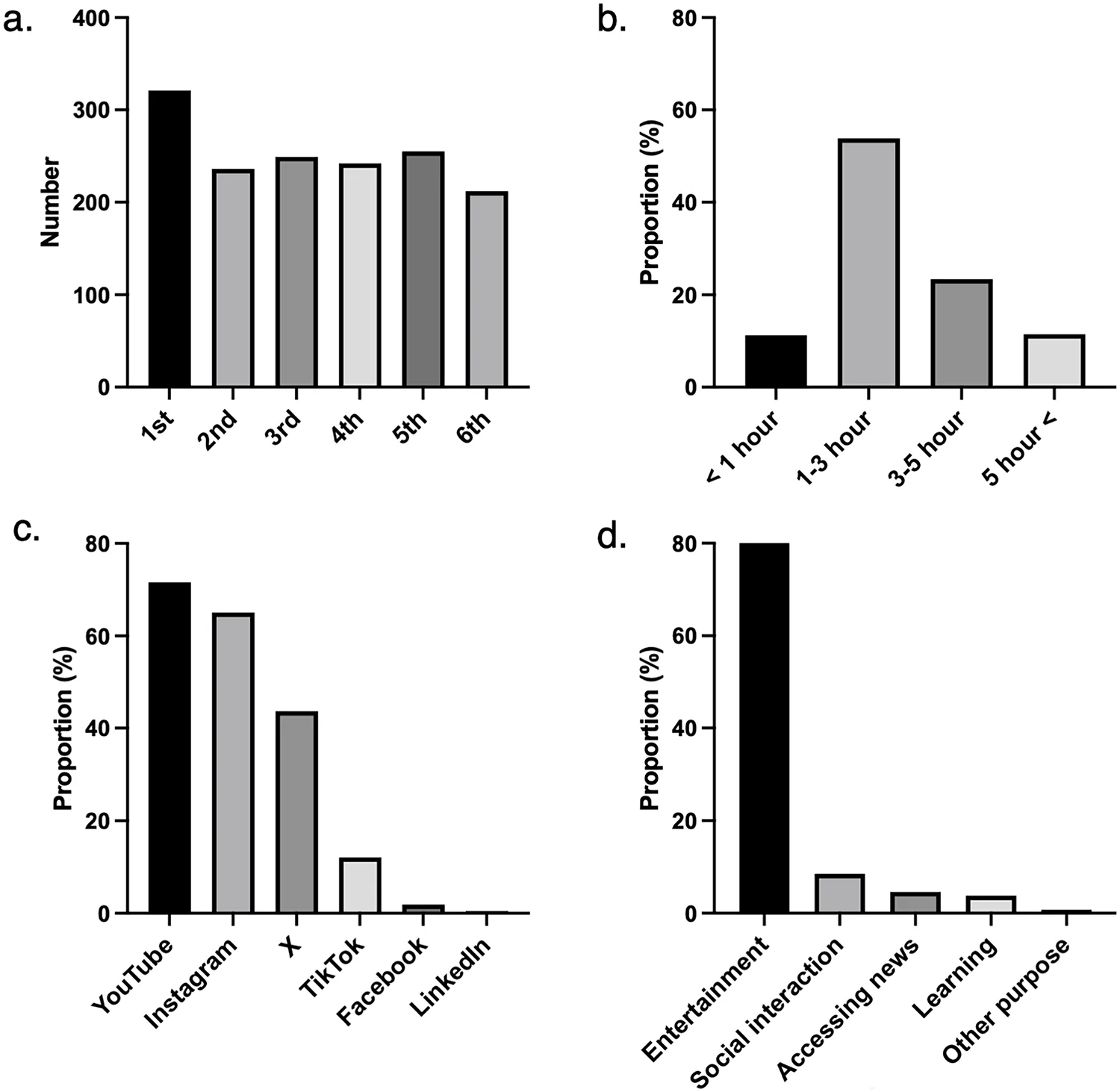
Overall characteristics of medical students’ social media use. (**a**) Number of respondents by year of study (first to sixth year), (**b**) Daily time spent on social media, (**c**) Overall proportion of medical students using each social media platform, (**d**) Primary purposes of social media use

A total of 1,515 medical students from eight universities were enrolled in this study. Table 1 summarizes their responses and Fig. 1a shows the number of respondents per year (first to sixth). Daily time spent on social media was most commonly reported as 1–3 h, followed by 3–5 h, while fewer students reported usage of < 1 h or > 5 h per day (Fig. 1b). As shown in Fig. 1c, YouTube (71.6%) was the most frequently used platform overall, followed by Instagram (65.1%) and X (43.7%). Conversely, few students reported using Facebook (1.9%) and LinkedIn (0.5%)..

**Table 1:** Participant characteristics and overall social media use among medical students

Characteristics (n=1515)	n (%)
Sex	
Male	861 (56.8)
Female	617 (40.7)
Prefer not to answer	37 (2.5)
Year of study	
1st year	321 (21.2)
2nd year	236 (15.6)
3rd year	249 (16.4)
4th year	242 (16.0)
5th year	255 (16.8)
6th year	212 (14.0)
Daily time spent on social media	
< 1 hour	171 (11.2)
1–3 hours	815 (53.8)
3–5 hours	355 (23.4)
≥ 5 hours	174 (11.5)
Most frequently used social media platforms*	
YouTube	1085 (71.6)
Instagram	987 (65.1)
X	662 (43.7)
TikTok	184 (12.1)
Facebook	29 (1.9)
LinkedIn	7 (0.5)
Primary purpose of social media use	
Entertainment	1248 (82.4)
Social interaction	129 (8.5)
Accessing news	69 (4.5)
Learning-related use	57 (3.8)
Other	12 (0.8)
Perceived usefulness of social media for learning	
YouTube	505 (33.3)
X	187 (12.3)
Instagram	64 (4.2)
TikTok	17 (1.1)
LinkedIn	10 (0.7)
Facebook	4 (0.3)
None	728 (48.1)
Perceived usefulness of social media for entertainment	
YouTube	695 (45.9)
Instagram	415 (27.4)
X	277 (18.3)
TikTok	99 (6.5)
Facebook	3 (0.2)
LinkedIn	2 (0.1)
None	24 (1.6)
Perceived usefulness of social media for social interaction	
Instagram	950 (62.7)
X	113 (7.5)
YouTube	11 (0.7)
TikTok	6 (0.4)
Facebook	10 (0.7)
LinkedIn	8 (0.5)
None	417 (27.5)
Perceived usefulness of social media for career decision-making	
Yes	763 (50.4)
No	752 (49.6)
Perceived as a useful platform for career decision-making	
X	255 (33.6)
YouTube	253 (33.4)
Instagram	225 (29.7)
TikTok	11 (1.5)
Facebook	10 (1.3)
LinkedIn	4 (0.5)

*Multiple responses were allowed for the most frequently used social media platforms

## Purposes of social media use

**Fig. 2:**
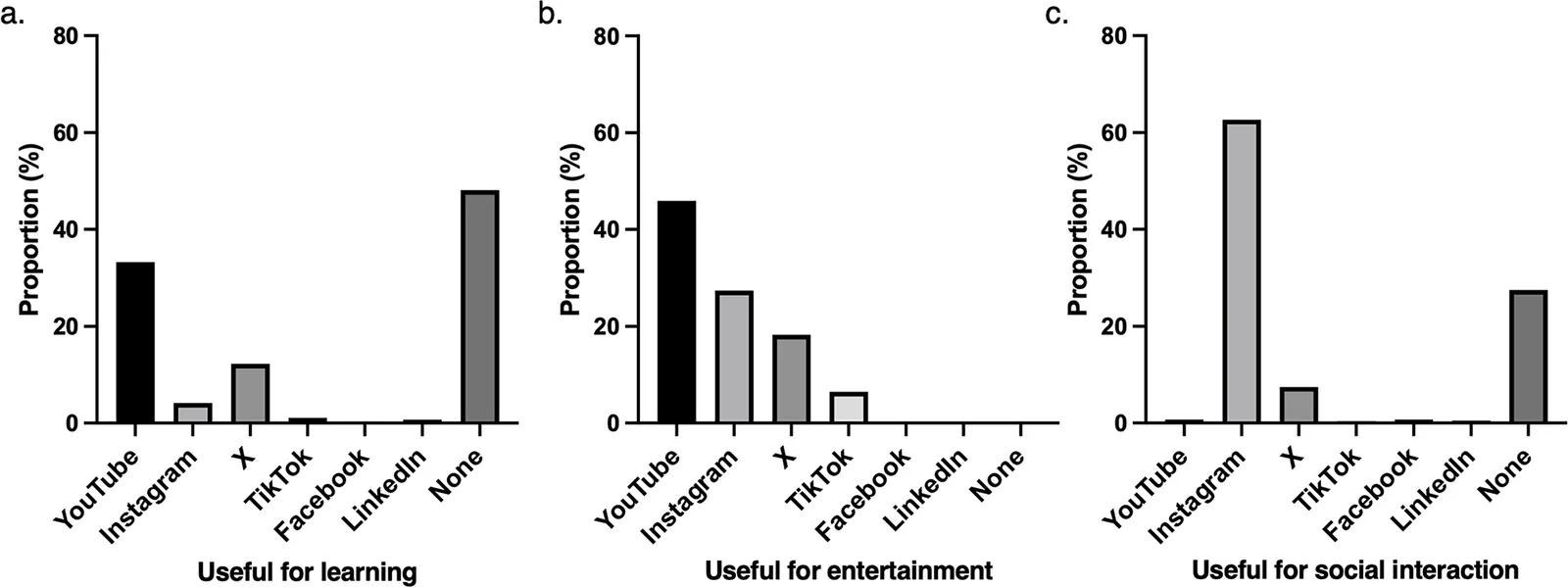
Social media platforms perceived as most useful for specific use. (**a**) Social media platforms perceived as most useful for entertainment purposes, (**b**) Social media platforms perceived as most useful for learning-related purposes, (**c**) Social media platforms perceived as most useful for social interaction purposes

Entertainment was the most frequently reported purpose of social media use, followed by social interaction, news access, and learning-related purposes (Fig. 1d). YouTube was the most commonly selected entertainment platform, followed by Instagram, X, and TikTok (Fig. 2a). YouTube was also the most frequently selected platform for learning purposes, followed by X and Instagram (Fig. 2b). In contrast, for social interaction, Instagram was by far the most commonly selected platform for social interaction, with minimal use of other platforms (Fig. 2c).

## Social media use by study year

**Fig. 3:**
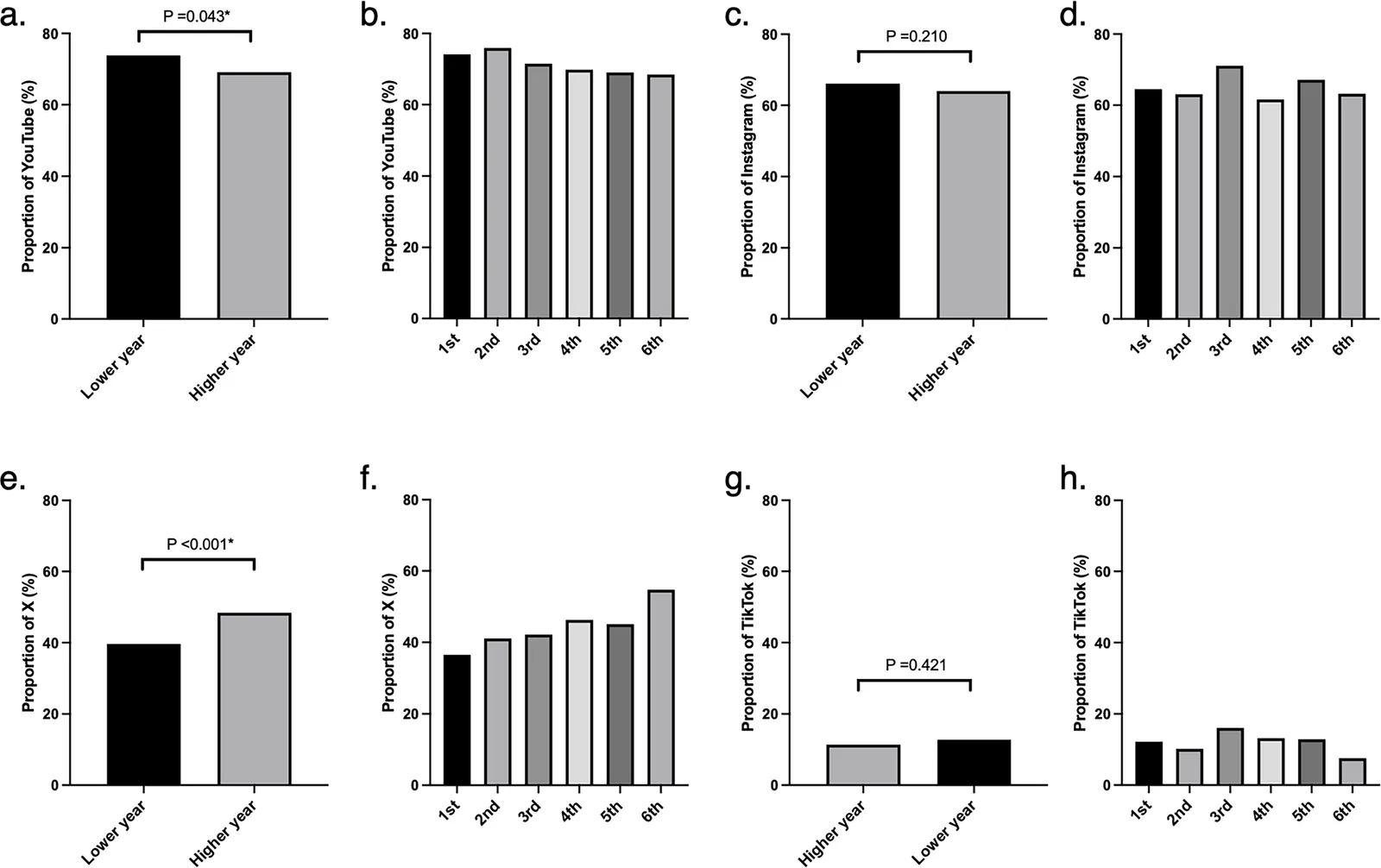
Differences in social media use by year of study. **(a**) Comparison of YouTube use between early-year and advanced-year medical students, (**b**) Proportion of students using YouTube by individual year of study, (**c**) Comparison of Instagram use between early-year and advanced-year medical students, (**d**) Proportion of students using Instagram by individual year of study, (**e**) Comparison of X use between early-year and advanced-year medical students, (**f)** Proportion of students using X by individual year of study, (**g**) Comparison of TikTok use between early-year and advanced-year medical students, (**h**) Proportion of students using TikTok by individual year of study

Figure 3 shows the differences in the usage of each social media platform by study year. Comparisons between early- and advanced-year students demonstrated significant differences in platform use for selected services. The proportion of students using YouTube was significantly higher among early-year students than among advanced-year students (*P* = 0.043) (Fig. 3a and b). In contrast, the use of X was significantly more common among advanced-year students (*P* < 0.001) (Fig. 3e and f). No significant differences were observed between groups for Instagram (*P* = 0.210) or TikTok (*P* = 0.421) (Fig. 3c, d and g, and 3h). When analyzed by individual year, the proportion of students using Instagram was consistently high across all years, whereas X use decreased among early-year students. The use of TikTok showed no specific patterns across all years.

### Perceived usefulness of social media for career decision-making

**Fig. 4:**
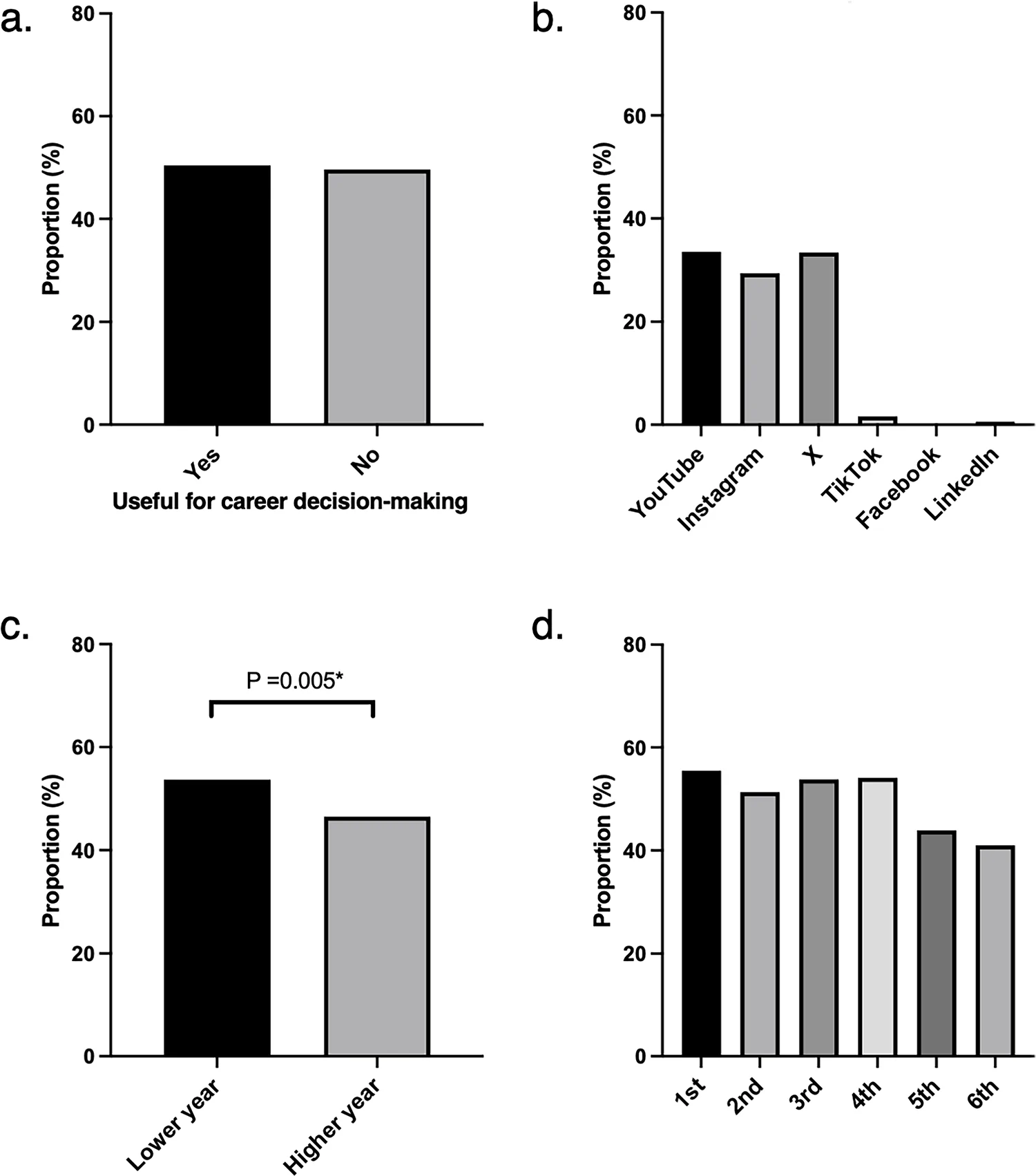
Perceived usefulness of social media for career decision-making. (a) Proportion of medical students reporting that social media is useful for career decision-making, (b) Social media platforms perceived as useful for career decision-making among students who answered “yes” in panel (a), (c) Comparison of perceived usefulness of social media for career decision-making between early-year and advanced-year students, (d) Proportion of students reporting perceived usefulness of social media for career decision-making by individual year of study

Figure 4 shows the perceived usefulness of social media for medical students’ career decision-making. A total of 763 respondents (50.4%) reported that social media was useful for career decision-making (Fig. 4a). These students considered X the most useful platform, followed by YouTube and Instagram (Fig. 4b).

Furthermore, early-year students were significantly more likely than advanced-year students to report that social media was useful for career decision-making (*P* = 0.005) (Fig. 4c). When analyzed by year of study, the proportion of students reporting perceived usefulness was comparable among first- to fourth-year students, but was notably lower among fifth- and sixth-year students (Fig. 4d).

## Discussion

This large-scale, multi-institutional survey provides a comprehensive overview of social media use among medical students in Japan. To our knowledge, this is one of the largest investigations of platform preferences, usage purposes, and perceptions of usefulness related to career decision-making among medical students. Our findings offer valuable insights for educators and clinicians seeking to effectively disseminate information to medical students via social-media platforms.

The present study identified that YouTube and Instagram were the most frequently used platforms among medical students, followed by X. Interestingly, the proportion of younger students using X decreased, whereas Facebook is no longer effective for reaching Japanese medical students, as only 1.9% reported using it. Kitamura et al. conducted a single-university survey on the social media usage of medical students and junior residents [13] and reported that the proportions of YouTube, Instagram, and X users were 85.1%, 71.3%, and 67.3%, respectively. The slightly higher proportion of X users reported in their study than in ours may be explained by differences in the study population, as their survey was limited to fourth- and fifth-year medical students and junior residents.

Social media-use patterns differ considerably across countries. A survey conducted in Dutch medical schools found that Facebook was the most popular platform, reported by more than 90% of medical students, followed by LinkedIn, with fewer than 10% using X [14]. Similarly, a survey conducted in France found that YouTube, Instagram, and Facebook were substantially more popular than X among medical students [15]. One possible explanation for these differences between Japan and other countries may be cultural variation in social media usage patterns. In Japan, many users tend to use platforms such as X and Instagram under pseudonyms, whereas the use of real names and identifiable profiles is more common in Western countries. Because Facebook generally requires the use of real names, this characteristic may make it less appealing than Instagram and X to some Japanese users especially in the young generations.

Notably, approximately half of the respondents reported that social media was useful for career decision-making. An important finding was that early-year students were significantly more likely to perceive social media as useful for this purpose than advanced-year students. The proportion was markedly lower among fifth- and sixth-year students than among students in the earlier years. This may be explained by the fact that students in their final years of medical school engage in clinical clerkships, during which they are exposed to abundant information through clinical environments and direct interactions with physicians, reducing their reliance on social media as an information source necessary for career decision-making. In this context, social media may complement students’ real-world clinical experience. Because clinical rotations often involve time constraints that limit the amount of teaching that can be delivered on the wards, social media platforms may provide opportunities to share supplementary educational content and maintain engagement with students outside the clinical environment. For example, our departments share educational posts based on the Japanese National Medical Licensing Examination to provide additional learning opportunities for medical students.

It is important to understand the differences in the social media platforms used by medical students according to their purpose of use. Interestingly, in this study, X was selected as the most useful platform for career decision-making. Unlike video-based platforms such as YouTube or image-centered platforms such as Instagram, X is primarily text-based and allows users to acquire a large volume of information within a limited time. This characteristic may make X particularly suitable for students seeking efficient access to career-related information. Furthermore, many academic journals have long disseminated scholarly content on their X accounts, including publications accompanied by visual abstracts and information related to academic conferences [16–20]. Consequently, X is likely to attract a substantial number of students with a strong interest in academic and scholarly information. This population is also more engaged in seeking information related to career decision-making.

Residency programs across various specialties have their own social media accounts, especially in the US. Previous studies have reported that 47–59% of general surgery residency programs in the US have social media accounts, with X and Instagram being the most commonly used platforms [5–7]. This trend accelerated during the COVID-19 pandemic when in-person education became challenging [7, 21]. Previous studies have examined the types of content shared on social media platforms. Although the majority of posts (70–81%) on general surgery residency programs were promotional in nature, educational content was associated with significantly higher engagement [22]. Platform use also differs across medical specialties. Facebook and X are used most frequently in vascular surgery [3]; X is most popular in pediatric surgery [11] and pathology [23]; and Instagram is most commonly used in obstetrics, gynecology [12], and plastic surgery [4].

It is equally important to understand which social media platforms are used by applicants when gathering information about residency programs. In a previous survey of ophthalmology residency applicants, 69% of the respondents reported using Instagram to obtain information about residency programs, and 58% indicated that social media influenced their decision to apply [9]. Similarly, most respondents in a survey of applicants to a general surgery residency program agreed that social media influenced their perception of the program positively, and helped them understand its culture and atmosphere better [10].

Accordingly, negative impressions of a specialty or an institution, if amplified on social media, may adversely affect recruitment. Therefore, professional societies and academic institutions should recognize that their official social media communications are utilized actively by students during career decision-making and adjust their dissemination strategies accordingly. From the perspective of recruiting staff, the social media accounts of prospective applicants may serve as an important source of information. Langenfeld et al. reported that program directors use applicants’ social media presence as part of their evaluations during the recruitment process [24]. Given this reality, medical students must be mindful of professionalism in online environments; so-called “online professionalism.” Failure to maintain appropriate professional behaviors on social media may have a negative impact on their future career opportunities.

Although findings from the US provide important insights into the role of social media in medical education and recruitment, it is unclear whether these findings apply to Japanese medical students. In Japan, an increasing number of surgical departments have begun using social media, particularly Instagram, to disseminate information to medical students and promote educational activities. However, there is currently a lack of evidence regarding which platforms are the most effective, which subgroups of students are being reached, and for what purposes students use social media in the Japanese setting. Therefore, our findings, which provide contemporary, large-scale evidence of social media use among medical students in Japan, are important. Future studies should investigate how engagement with social media influences medical students’ specialty preferences. In particular, understanding whether and how social media exposure affects interest in surgical careers may help design more effective strategies for attracting the next generation of surgeons. Furthermore, we should examine social media use among residents and early-career physicians to identify whether the patterns observed in medical students persist during postgraduate training and whether social media engagement influences career satisfaction or subspecialty choices.

The present study has several limitations. First, although the survey included both public and private medical schools and covered a wide geographical range, it was limited to eight institutions. Therefore, the generalizability of these findings to all medical students in Japan or other countries may be limited. Second, the use of a self-administered questionnaire introduces the possibility of response bias, including recall and social desirability biases. Finally, platform usage was assessed primarily through self-reported frequency and perceived usefulness without objective measures of actual engagement or content quality. Moreover, this survey did not assess the specific types of social media content that may influence medical students’ career decision-making. Future studies should investigate what content, such as posts related to clinical training, physicians’ daily work, or educational activities, is most strongly associated with students’ specialty preferences.

It is important to note that social media platforms used by students are likely to evolve rapidly. Although TikTok usage was relatively low in the present survey, trends toward short-form video content, such as YouTube Shorts and Instagram Reels, suggest that preferences may shift in the near future. Consequently, the findings of this study should be interpreted as reflecting platform usage patterns at a specific point in time: 2025.

Despite its limitations, this study represents one of the largest surveys examining social media use among medical students and provides findings that may be useful for educators and clinicians involved in medical student education and recruitment.
