# Correction: How do Japanese medical students use social media? A large multicenter survey of platform preferences and career decision-making

**DOI:** 10.1007/s00595-026-03464-0

**Published:** 2026-08-10

**Authors:** Takehito Yamamoto, Koya Hida, Shin Ishihara, Soichi Murakami, Yusuke Yamane, Tatsuya Kinjo, Misako Kinoshita, Kenta Horimukai, Atsushi Otsuka, Hideki Takami, Kazutaka Obama

**Affiliations:** 1 Department of Surgery, Graduate School of Medicine, Kyoto University, 54 Shogoin- Kawahara-cho, Sakyo-ku,, Kyoto, 606-8507 Japan; 2 Department of Gastroenterological Surgery, Fujita Health University, Toyoake, Japan; 3 Department of Gastroenterological Surgery II, Graduate School of Medicine, Hokkaido University, Sapporo, Japan; 4 Department of Surgical Oncology, Graduate School of Biomedical Sciences, Nagasaki University, Nagasaki, Japan; 5 Department of Digestive and General Surgery, Faculty of Medicine, University of the Ryukyus, Okinawa, Japan; 6 Department of Pediatrics, Jikei University Katsushika Medical Center, Katsushika, Japan; 7 Department of Dermatology, Kindai University Hospital, Osaka, Japan; 8 Department of Surgery, Graduate School of Medicine, Nagoya University Hospital, Nagoya, Japan

**Correction to: Surgery Today (2026)**

**https://doi.org/10.1007/s00595-026-03328-7**

In the original publication, in the Results section, the following sentence was published incorrectly:

“Conversely, few students reported using Facebook (1.9%) and LinkedIn (0.8%).”

The correct sentence is:

“Conversely, few students reported using Facebook (1.9%) and LinkedIn (0.5%).”

This value is consistent with Table 1, which correctly reports the number of LinkedIn users as 7/1,515 (0.5%). This correction is purely typographical and does not affect the analyses, results, interpretation, or conclusions of the study.

The original article has been corrected.

